# Innate Host Habitat Preference in the Parasitoid *Diachasmimorpha longicaudata*: Functional Significance and Modifications through Learning

**DOI:** 10.1371/journal.pone.0152222

**Published:** 2016-03-23

**Authors:** Diego F. Segura, Ana L. Nussenbaum, Mariana M. Viscarret, Francisco Devescovi, Guillermo E. Bachmann, Juan C. Corley, Sergio M. Ovruski, Jorge L. Cladera

**Affiliations:** 1 Laboratorio de Genética de Insectos de Importancia Económica, IGEAF, CICVyA, INTA, Hurlingham, Argentina; 2 Consejo Nacional de Investigaciones Científicas y Técnicas (CONICET), Buenos Aires, Argentina; 3 Insectario de Investigaciones para Lucha Biológica, IMyZA, CICVyA, INTA, Hurlingham, Argentina; 4 Grupo de Ecología de Poblaciones de Insectos. INTA EEA Bariloche, San Carlos de Bariloche, Argentina; 5 PROIMI Biotecnología, CCT Tucumán CONICET, División Control Biológico de Plagas, San Miguel de Tucumán, Argentina; Rutgers University, UNITED STATES

## Abstract

Parasitoids searching for polyphagous herbivores can find their hosts in a variety of habitats. Under this scenario, chemical cues from the host habitat (not related to the host) represent poor indicators of host location. Hence, it is unlikely that naïve females show a strong response to host habitat cues, which would become important only if the parasitoids learn to associate such cues to the host presence. This concept does not consider that habitats can vary in profitability or host nutritional quality, which according to the optimal foraging theory and the preference-performance hypothesis (respectively) could shape the way in which parasitoids make use of chemical cues from the host habitat. We assessed innate preference in the fruit fly parasitoid *Diachasmimorpha longicaudata* among chemical cues from four host habitats (apple, fig, orange and peach) using a Y-tube olfactometer. Contrary to what was predicted, we found a hierarchic pattern of preference. The parasitism rate realized on these fruit species and the weight of the host correlates positively, to some extent, with the preference pattern, whereas preference did not correlate with survival and fecundity of the progeny. As expected for a parasitoid foraging for generalist hosts, habitat preference changed markedly depending on their previous experience and the abundance of hosts. These findings suggest that the pattern of preference for host habitats is attributable to differences in encounter rate and host quality. Host habitat preference seems to be, however, quite plastic and easily modified according to the information obtained during foraging.

## Introduction

Host plants are a major source of information for insect parasitoids during host searching [1–3], even when they are not infested [4–6]. The damage caused by the herbivore can induce the plants to release specific compounds (termed herbivore-induced plant volatiles) that increase the attraction of parasitoids [7–9]. Communication between the first and third trophic level is expected to be particularly enhanced when the herbivores are concealed within the host plant, thus reducing to a minimum the available information about their location [10].

The type of chemical cues used by parasitoids during host searching has been related by Vet and Dicke [11] to the range of plant species where the host can be found. When the hosts are generalists, female parasitoids should rely on general cues, common to all possible host plant species, and would follow specific cues only after they acquire experience and associate conspicuous cues (mainly from the plant) to the host presence, through associative learning [12–15]. However, Steidle and van Loon [16] found many examples of parasitoids of generalist hosts that innately respond towards chemical cues from the host habitat. Vet and Dicke’s [11] concept does not consider that different host plants species may differ in their profitability (host density and/or encounter rate) as well as in the quality of the hosts they harbour [17–19]. In fact, if host availability differs among host plants, optimal foraging theory [20,21] predicts that females should always prefer the most profitable host plant. Therefore, an innate response towards chemical cues could have evolved from some host-plant systems if the reward in terms of cumulative fitness varies among them [22]. Likewise, if the nutritional quality of the herbivore host varies among plant species, natural selection should favour female parasitoids that are attracted to plants in which the hosts are nutritionally better [23–26], a concept known as the preference–performance hypothesis (PPH) [27,28]. PPH has been widely studied in bi-trophic scenarios: host plant-herbivore (see Gripenberg et al. [28] and references herein) and parasitoid-herbivore host [29–31].

Previous studies have shown that the plant species on which the herbivores develop influences parasitoid’s offspring performance [32–35]. Furthermore, there are robust evidences that host plant species affect the second and third trophic levels simultaneously [36–41]. Concurrently, several studies on parasitoids reported a preference for particular host habitats [42–44]. Nonetheless, there is a limited number of studies that simultaneously addressed preference and performance considering the first and third trophic level [45], and are mainly focused on parasitoids associated to Brassicaceae species [45–47] or to Tephritidae fruit fly species [48,49].

*Diachasmimorpha longicaudata* (Ashmead) is a koinobiont endoparasitoid of Tephritidae larvae. In its habitat of origin, *D*. *longicaudata* parasitizes larvae of several species of the genus *Bactrocera* [50,51], while they are feeding inside the fruit. Most of its hosts are polyphagous (for instance *Bactrocera dorsalis* Hendel attacks more than 150 fruit species), attacking several unrelated families of plants [51,52], including (Caricaceae, Moraceae, Myrtaceae, Rosaceae, and Solaneaceae, among the most important). Because larvae develop entirely in the same fruit, fruit species may be a reliable indicator of the quality of the larvae as hosts. Fruit species could also vary in the host encounter or parasitization rates, so the costs (energy and time allocated to host searching) would differ among host fruits [30]. These differences in potential rewards could have led to host fruit preference. In fact, innate preference for fruit species has been reported for this species [53,54]. Nonetheless, the evolutionary forces that forged these preference patterns have seldom been addressed because [as stated by 45] preference and performance associated to different host habitats have been studied separately [33,55,56] except for Eben et al. [48] and Ovruski et al. [57] who found no preference for fruit species that differentially affected female performance.

Parasitoids of polyphagous hosts, such as *D*. *longicaudata*, should profit from adjusting their foraging preferences to the distribution of their hosts. Associative learning has been documented for fruit fly parasitoids [58,59], including *D*. *longicaudata* [60], however its role in modulating the host habitat preference has not been addressed. Likewise, the fact that the density of host larvae varies among different fruits [61,62] has not been considered.

In this work, we addressed the innate preference of *D*. *longicaudata* females among odours from four host habitats and, when a preference pattern was found, we tested both the PPH and the potential benefits in terms of parasitization rate. We also tested the hypothesis that *D*. *longicaudata* is able to adjust host habitat preferences as a function of the density of hosts in the available habitats. Finally, because *D*. *longicaudata* is capable of associative learning during host finding [60], we hypothesized that the preference for chemical cues of available fruit species is modified by experience to the point where innate preference will no longer drive parasitoid behaviour.

## Methods

### Insects and fruits

Parasitoids and fruit fly larvae were obtained from the rearing facility at Instituto de Genética “E. A. Favret” (IGEAF) [63]. *Diachasmimorpha longicaudata* colony was initiated with individuals coming from Centro de Investigaciones para la Regulación de Poblaciones de Organismos Nocivos (CIRPON), Argentina [64] in 2001. *Ceratitis capitata* (Wiedemann) (Diptera: Tephritidae) larvae were used as host. Larvae were reared on artificial rearing medium (a mixture of carrot, sugar, brewer’s yeast, corn flour, and food preservatives, according to Terán [65]) and were exposed to parasitoid females in small Petri dishes when they reached the third instar (following Viscarret et al. [63]). Assays were carried out with 5–7 days-old females with no oviposition experience (except in experiment 3). Females were maintained with males under controlled conditions (25 ± 1°C, 65 ± 5% R.H., and 14:10 L:D photoperiod), and were provided with honey and water ad libitum. Females had no contact with fruit or fruit odours until the test. Fruit showing no signs of insect infestation were obtained from the local market. All fruit were thoroughly washed with tap water, measured and maintained at 25 ± 1°C and 50 ± 5% R.H. until the tests.

### Y-tube olfactometer

The olfactory preferences of *D*. *longicaudata* were tested using a Y-tube glass olfactometer, in which the females cannot see the fruit and they only perceive chemical cues. The air flow inside the Y-tube was generated by extracting air with a pump (AIR CADET Barnat, USA) connected to the end of the device. The air entered the system through a pair flowmeters (Bruno Schilling, Argentina) that were set at 300 ml/min (0.014 m/s). After entering the device the air was filtered (with glass wool and activated charcoal) and then bubbled in distilled water. Subsequently, the air stream entered two acrylic boxes (3.375 L) where the odour sources were held. Acrylic boxes were connected to the Y-tube which consisted in a two 15 cm-long arms (60° angle between arms, internal diameter 3cm) converging in a 15 cm-long central tube. The distal extreme of the central tube has a hole to introduce the insect. All parts were connected using colourless and odourless plunges and tubing (Dow Corning, Midland, Michigan, USA). The Y-tube assembly was illuminated by a fluorescent light tubes that provided a homogenous illumination of 1200 ± 100 luxes. The room was maintained at 26 ± 1°C and 70 ± 5% R.H.

Females were individually released inside the tube and only after they showed signs of acclimatization (i.e., exhibited host searching behaviours such as antennation) the air pump was turned on. Insects were given 10 min to choose an arm in the olfactometer. It was considered that a female made a choice when it walked into one of the arms, surpassed a distance of 4 cm from the end of the central tube and stayed beyond that limit for more than 30 s. The chosen arm and the latency (time since the release to the decision) were recorded. If no choice was made in 10 min, the assay was concluded.

Every five tested females, the Y-tube and the acrylic boxes were cleaned with hot water, rinsed with ethanol, and left to air dry at room temperature. With each new experimental series of five females, the location of the odour sources was switched in the opposite arrangement. Forty females were evaluated for each treatment.

### Experiments

#### Experiment 1. Preference of *D*. *longicaudata* females for different fruit species

Four fruit species were tested: peach (*Prunus persica* L., variety Elegant Lady), fig (*Ficus carica* L., variety Brown Turkey), orange [*Citrus sinensis* (L.) Osbeck, variety Valencia], and apple (*Malus domestica* Borkh, variety Red Delicious). These species represent three of the most important taxonomic families where *Bactrocera* larvae can be found in nature [52]. Fruit were offered to female parasitoids in every possible pair-wise combination. Forty females (replicates) were analysed per combination. Each acrylic box contained one apple, one peach, one orange or three figs. This way the amount of surface of each fruit was as similar as possible.

#### Experiment 2. Effect of the fruit infestation level on females’ preference for host habitats

For this set of experiments, apple and orange were selected as study models and female choice was evaluated in four different scenarios: a) orange with high infestation vs. orange with low infestation; b) apple with high infestation vs. apple with low infestation; c) orange with high infestation vs. apple with low infestation; and d) apple with high infestation vs. orange with low infestation. Forty females (replicates) were analysed per combination. This pair of fruits was used as model based on the result of experiment 1 and the fact that are available in the Argentinean market throughout the year (opposite to fig and peach which are available for 1 and 3 months, respectively).

Infested fruit were obtained by placing 60 *C*. *capitata* females inside a rearing cage (64 L) with four fruit for 1 hour. After infestation, fruit was placed individually in plastic containers (1 L) and kept under controlled conditions (25 ± 1°C; 40 ± 5% R.H.). To estimate the infestation level without damaging the fruit, the number of larvae that exited the fruit was recorded the day after the first emerging larvae were detected. If one or two larvae exited the fruit within this 24h time frame, the fruit was assigned to the low infestation level; whereas if 5–10 larvae were recorded the fruit was assigned to the high infestation level. After the assay, the fruit were labelled and kept individually until all larvae emerged and could be counted. Highly infested fruits should have had 30–40 inside at the time of the experiment, whereas fruits with low infestation should have had 5–10 larvae. This procedure allowed obtaining infestation levels that resembled low and high infestation in nature. [66]. Those experimental series (i.e., five females) for which the infestation estimation method was not effective for predicting larval abundance, were removed from the dataset.

#### Experiment 3. Effect of previous experience on females’ preference for host habitats

Host habitat preference was assessed using females that had been offered host larvae associated to a specific habitat during a conditioning period. Again, orange and apple were used as experimental models. Conditioning protocol followed Segura et al. [60], which proved effective to condition *D*. *longicaudata* females to a visual stimulus. During the conditioning period, 15 females were offered a small Petri dish (5 cm in diameter, 1 cm high, wrapped in *voile* fabric) containing 100 larvae of *C*. *capitata* immersed in orange or apple pulp for 6h. Exposed larvae were kept under controlled conditions (25 ± 1°C; 65 ± 5% R.H.) until adult emergence as to check that females had successfully parasitized them during the conditioning period. The procedure was repeated for three consecutive days. On the fourth day, conditioned females were used in olfactometry assays where preference for orange or apple was assessed. Forty females (replicates) were analysed per combination.

#### Experiment 4. Quality of different fruit species as foraging substrates

In order to compare the foraging efficiency of *D*. *longicaudata* in different host habitats, infested oranges, apples, figs and peaches were exposed to naïve parasitoids. Infestation was achieved as described in experiment 2 (except for fig, in which 12 fruit were exposed to 60 *C*. *capitata* females). Fruit was then measured and placed in individual containers. When the 3^rd^ larval instar was reached, fruit were individually exposed to *D*. *longicaudata* females for 6 h. Oranges, apples and peaches were exposed to 5 female parasitoids, whereas figs were exposed to 3 female parasitoids. This proportion allowed standardizing the area offered to the females (ca. 25 cm^2^/female) among different fruit species. 120 replicates were carried out for each fruit species.

After exposure to parasitoids, fruit were placed in individual containers and kept under controlled conditions (25 ± 1°C; 40 ± 5% R.H.). Pupae were collected, counted, individually weighed to the nearest 0.1 mg using a precision scale (Denver Instrument, NY, USA). Afterwards, pupae were transferred to glass containers and maintained under controlled conditions (26 ± 1°C; 70 ± 10% R.H.) until adult (parasitoids or flies) emergence. The number of flies and parasitoids was recorded and the non-emerged pupae were dissected under a stereomicroscope to determine if the content corresponded to a fly or a parasitoid.

#### Experiment 5. Quality of host larvae reared on different fruit species

Several bionomic parameters were evaluated on parasitoids emerging from experiment 4. To this end, a female and a male parasitoid emerged from larvae developed in the same fruit species were placed in glass flaks (500 ml) with water and honey, and kept at 22 ± 1°C, 60 ± 5% R.H. and a 14:10 L:D photoperiod.

The mortality was recorded daily. Whenever a male died, it was replaced by another male to ensure continuous availability of sperm. One week after female emergence, 70 3^rd^ instar *C*. *capitata* larvae, confined in small Petri dishes, were exposed to females for 6h. For each female, 9 larval exposures were performed every 48–72 h covering the period of higher fecundity [63]. Exposed larvae were transferred to a vial containing fresh artificial larval medium which in turn was placed inside a larger container with a layer of vermiculite and kept under controlled conditions (25 ± 1°C; 70 ± 5% R.H.). Every 48h, pupae were collected and counted, and then placed in glass flasks (450 ml). The number of emerged flies and parasitoids (males and females) was recorded. Non emerged pupae were dissected under a stereomicroscope to determine if the content corresponded to a fly or a parasitoid. For apple and orange, 40 couples (replicates) were evaluated, whereas 36 and 30 replicates were carried out for fig and peach, respectively.

Dead females were preserved in ethanol 70%. After all female died, a random sample was taken from each group (28, 32, 18 and 15 for apple, orange, fig and peach respectively) and the right front wing of each female was dissected and placed on a slide over a thin layer of silicone. Wings were photographed under a stereomicroscope (Motic Group Co., China) and the length of the wing was measured afterwards using Motic Images Plus 2.0 software (Motic Group Co., China).

### Data analysis

In experiments 1 to 3, a G-test of goodness of fit (with Yates’ correction for continuity) was used to compare the frequency of insects visiting each option. Latency was compared between options by means of a Student *t*-test (assumptions were met in all cases).

In experiment 4, parasitism rate was estimated as: (total number of parasitoids / total number of recovered pupae) x 100. For this rate, emerged and non-emerged insects (flies and parasitoids) were considered. This variable was compared by means of a one-way ANOVA, followed by a post hoc Tukey’s multiple comparisons test. As to address whether the different fruit species offered the same possibility of finding host larvae, the number of pupae recovered per fruit per unit area was compared by means of a one-way ANOVA and a post hoc Tukey’s multiple comparisons test after the natural log transformation.

In experiment 5, the mean fecundity (number of offspring per female in each exposition), the total number of offspring per female (lifetime fecundity), the sex ratio (number of females in the progeny/ total number of offspring) and female right wing length were compared by means of a one-way ANOVA, followed by a post hoc Tukey’s multiple comparisons test. Sex ratio was previously transformed to logit to meet ANOVA assumptions. The weight of *C*. *capitata* pupae recovered from different fruit species was compared through the estimation of the mean and the confidence intervals. For this variable, severe heteroscedasticity impeded to run an ANOVA or even a Kruskal-Wallis test, as no transformation allowed reducing differences in variance among treatments. Heteroscedasticity was due to a significantly larger variation in weight of pupae recovered from fig, so an exploratory ANOVA (followed by a post hoc Tukey’s multiple comparisons test) was carried out with the remaining fruit species.

Longevity was compared among treatments by means of a Cox-Mantel survival analysis. Survival curves were computed using the Kaplan-Meier method.

Tests were performed using STATISTICA for Windows [67].

## Results

### Experiment 1. Attraction of *D*. *longicaudata* females’ to different fruit species

**Fig 1:**
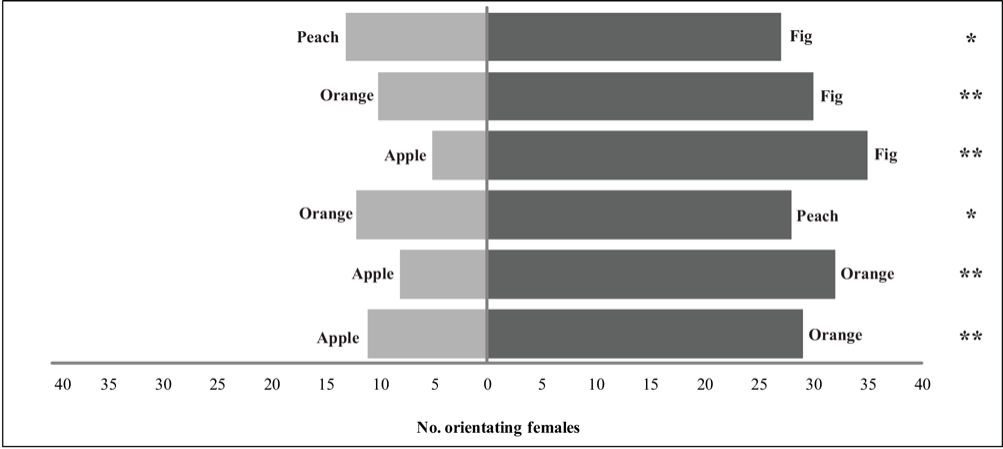
Preference for different fruit species by *Diachasmimorpha longicaudata* females in a Y-tube olfactometer. Bars show the number of females visiting each option in each pair-wise combination among the four fruit species (experiment 1). G-test level of significance: * p < 0.05; ** p < 0.01.

Females showed a clear preference for figs compared to the other fruit (peach, orange and apple) (G_fig-peach_ = 4.30, p = 0.038; G_fig-orange_ = 9.40; p < 0.01; G_fig-apple_ = 23.42; p < 0.01). Peach was preferred over orange and apple (G_peach-orange_ = 5.76, p = 0.016; G_peach-apple_ = 14.07, p < 0.01). Finally, females showed a preference for orange over apple (G_orange-apple_ = 7.46, p < 0.01).These results showed a hierarchical pattern of female preference for different fruit species. The most attractive fruit was fig, then peach, then orange and finally apple (Fig 1). There were no significant differences in latency between options, except for the fig that was chosen more quickly than the orange (S1 Table).

### Experiment 2. Attraction of *D*. *longicaudata* females’ to fruit with different levels of infestation

**Fig 2:**
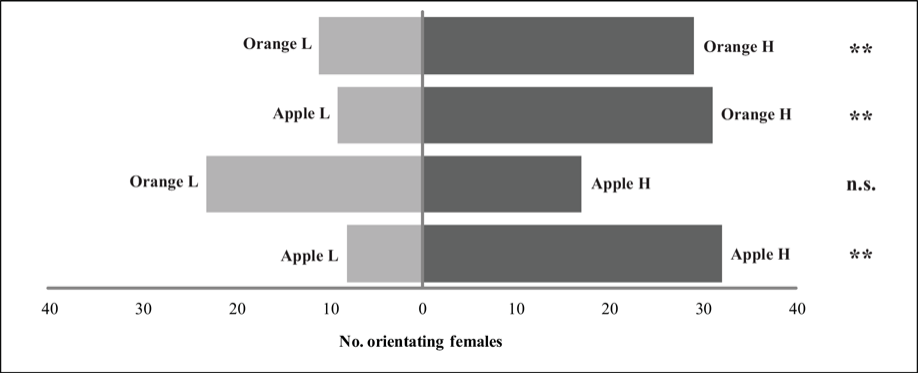
Preference of *Diachasmimorpha longicaudata* females for fruit with different levels of infestation by *Ceratitis capitata*. Bars show the number of females visiting each option in the experiment 2. H: high infestation level; L: low infestation level. G-test level of significance: n.s. = non-significant; * p < 0.05; ** p < 0.01. n.s.

When fruit of the same species were offered to the females in the Y-tube, a clear preference for the most infested fruit was shown (G_apple high-apple low_ = 14.32, p < 0.01; G_orange high-orange low_ = 7.46, p < 0.01) (Fig 2). Preference for the most infested fruit was also found when females were offered an orange with high infestation and an apple with low infestation (G_apple low-orange high_ = 11.59, p < 0.01) (Fig 2). However, apples with high levels of infestation were not more attractive than oranges with low levels of infestation (G_apple high-orange low_ = 0.63, p = 0.428) (Fig 2). Latency times did not differ between treatments (S2 Table).

### Experiment 3. Effect of previous experience on *D*. *longicaudata* females’ preference

**Fig 3:**
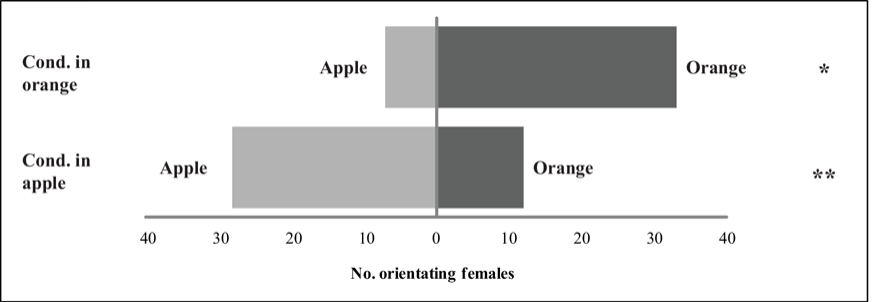
Effect of previous experience on the preference of *Diachasmimorpha longicaudata* females for different fruit species. Bars show the number of females (conditioned either on apple or orange) visiting each option in the experiment 3. G-test level of significance: * p < 0.05; ** p < 0.01.

In the Y-tube olfactometer, a significantly higher proportion of females oriented towards the fruit on which they were conditioned, irrespectively of the fruit species (Females conditioned on orange: G_apple -orange_ = 16.85, p < 0.01; females conditioned on apple: G_apple -orange_ = 5.76, p = 0.016) (Fig 3). Latency times did not differ between treatments (S3 Table). Parasitism controls showed that all the females used in experiment 3 had successfully parasitized larvae during the conditioning period.

### Experiment 4. Quality of different fruit species as foraging substrates

While the number of fruit exposed to parasitoids was 120 for each fruit species, several replicates were lost for different reasons. In some cases, larvae were not recovered from some fruit that were considered to be infested (and were consequently offered to parasitoids). Likewise, some fruit were colonized by fungi and larval survival was severely affected. These cases were not included in the dataset and consequently the number of replicates was 295 (Apple: 58 fruit; Fig: 58 fruit; Orange: 64 fruit; Peach: 115 fruit).

**Fig 4:**
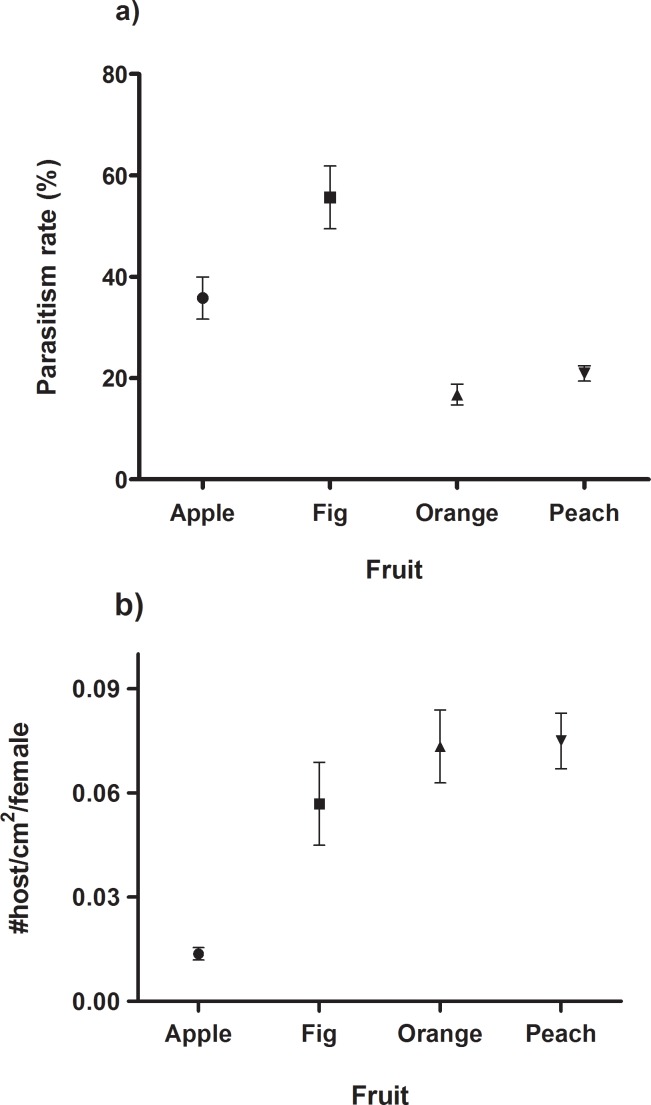
Quality of different fruit species as foraging substrates. a) Mean parasitism rate by *Diachasmimorpha longicaudata* recorded in different fruit species infested by *Ceratitis capitata* larvae. b) Mean density of larvae offered during infested fruit exposure in experiment 4. Bars show the standard error of the mean.

The parasitism rate ranged between 16.76% and 55.66%, and significantly differed among fruit species (ANOVA: F_3, 291_ = 28.43; p < 0.01). Tukey’s multiple comparisons showed that the parasitism rates in fig were higher than in apple, orange and peach (Fig 4A). In turn, apple showed higher rates than orange and peach, which showed no differences between them (Fig 4A).

The number of larvae per cm^2^ of fruit surface offered to female parasitoids was significantly different among fruit species (ANOVA: F_3, 291_ = 22.74; p < 0.01) (Fig 4B). Multiple comparisons post hoc test showed that apple had significantly lower density of larvae than the other fruit species (Fig 4B), whereas no differences were found among the other three species (Fig 4B).

### Experiment 5. Quality of host larvae reared on different fruit species

**Fig 5:**
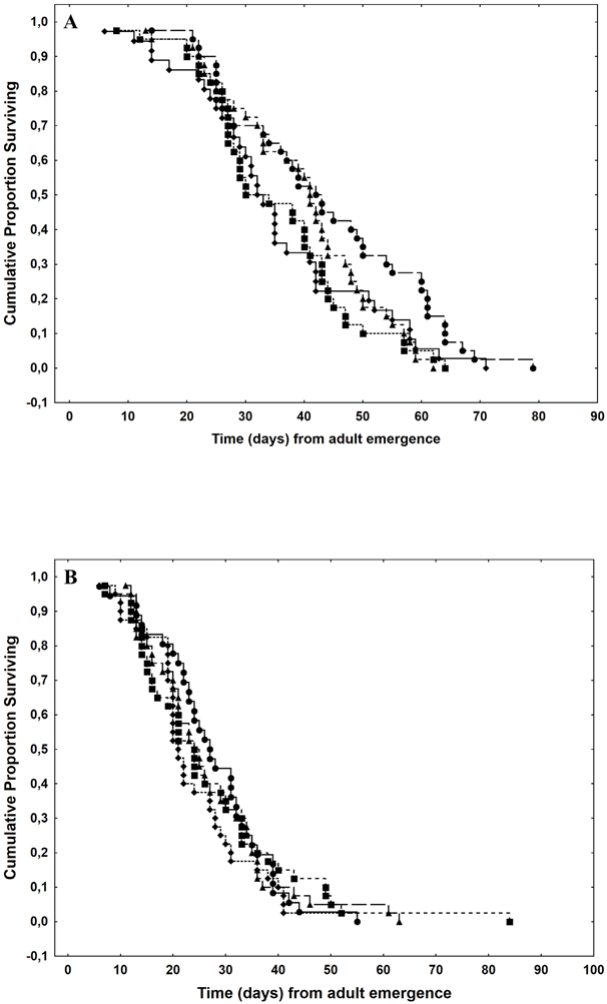
Survival curves for adult *Diachasmimorpha longicaudata* reared on *Ceratitis capitata* larvae that developed in different host fruit. Different fruit species are labelled with a different shape: Apple: circles; Fig: squares; Orange: triangles; Peach: diamonds. (a) males and (b) females.

Mean daily fecundity ranged between 13.14 and 19.41 parasitoids/female and the total fecundity ranged between 106.13 and 160.12 parasitoids/female. Significant differences were found in the mean fecundity of parasitoid emerged from larvae that had fed on different fruit species (ANOVA: F_3, 142_ = 32.08, p < 0.01) (Table 1). The mean fecundity of females emerged from larvae reared on oranges was higher than the other fruit species, which in turn did not differ statistically (Table 1). The same pattern was observed for the lifetime fecundity (ANOVA: F_3, 142_ = 31.80, p < 0.01; Table 1). The sex ratio differed significantly among treatments (ANOVA: F_3, 142_ = 6.26, p < 0.01). Females reared on larvae that developed in oranges showed a higher proportion of females in the progeny than females reared in the other three fruit species, which showed no differences among them (Table 1). Survival analyses showed no differences in lifespan among treatments, both for females and males (Cox-Mantel test: Males: Chi^2^_3_ = 2.81, p = 0.421; Females: Chi^2^_3_ = 6.28, p = 0.099) (Table 1, Fig 5).

**Table 1:** Mean fecundity (mean ± S.E.), total fecundity (mean ± S.E.), sex ratio (mean ± S.E.) and lifespan (mean ± S.E., in days) of *Diachasmimorpha longicaudata* reared in fruit fly larvae fed on different fruit species in experiment 5.

Fruit species	Mean fecundity	Total fecundity	Sex ratio	Female lifespan	Male lifespan
Apple	13.14 ± 1.03 a	106.13 ± 10.11 a	0.477 ± 0.019a	41.25 ± 2.62	26.56 ± 1.94
Fig	15.89 ± 1.23 a	128.89 ± 10.87 a	0.500 ± 0.026a	35.19 ± 2.56	27.50 ± 1.78
Orange	19.41 ± 1.11 b	160.12 ± 13.79 b	0.646 ± 0.021b	35.17 ± 2.03	24.08 ± 1.64
Peach	14.18 ± 0.90 a	125.18 ± 10.68 a	0.531 ± 0.029a	39.32 ± 2.04	26.80 ± 2.42

Means followed by the same letter within the same column do not differ statistically (α = 0.05; multiple contrasts).

The weight of pupae reared in each fruit species was higher for peach and orange; fig showed intermediate values, and apple showed the lowest values (Table 2). The differences among the four fruit species could not be analysed statistically, and the ANOVA performed excluding the fig showed significant differences among the other three species (ANOVA: F_2, 234_ = 54.78, p <0.01). Post hoc comparisons showed that pupae that developed in apple were lighter than pupae that developed in orange and peach, between which there were no significant differences in weight (Table 2).

**Table 2:** Pupal weight (mean ± S.E.) of *Ceratitis capitata* reared on different fruit species.

Fruit species	Pupal weight (mg)	CI 95%
Apple	7.09 ± 0.17a	6.74–7.43
Fig	8.46 ± 0.36	7.74–9.19
Orange	9.16 ± 0.15b	8.86–9.45
Peach	9.34 ± 0.14b	9.06–9.62

Confidence intervals of 95% (CI 95%) are also presented.

Means followed by the same letter within the same column do not differ statistically (α = 0.05; multiple contrasts). Fig was not considered in the statistical analysis because of the larger variation around the mean.

**Fig 6:**
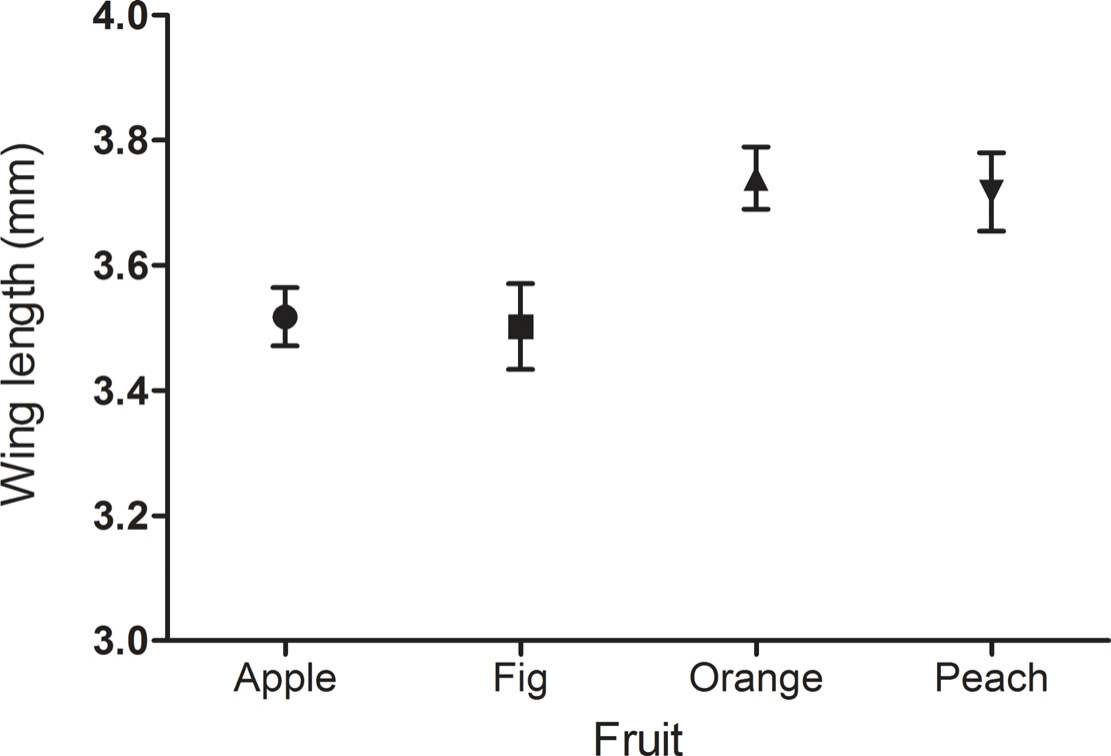
Effect of fruit species on parasitoid size. Mean wing length of *Diachasmimorpha longicaudata* reared on *Ceratitis capitata* larvae that developed in different host fruit. Bars show the standard error of the mean.

Wing length was statistically different among females emerged from larvae reared in different fruit species (ANOVA: F_3, 89_ = 5.37, p < 0.01). Tukey’s test showed that wings from females associated to oranges were larger than those associated to figs (p = 0.017) and apples (p < 0.01), but no differences were found among figs, apples and peaches (p > 0.05) (Fig 6).

## Discussion

Innate preference for host habitats in parasitoids is expected to occur only if available habitats vary in profitability and/or host quality. In our study, naive *D*. *longicaudata* females showed a hierarchical preference pattern among non-infested fruit of four different species. This result can be partially explained by a differential reward in terms of fitness associated to each fruit species. Evidence show that the pattern of preference is not fixed and can be modified according to external factors, such as host density, and internal factors, such as experience.

Females preferred fig over the rest of the fruit species, then peach, orange and, finally, apple. This innate preference was not expected to be so strong for a parasitoid which hosts are so polyphagous. The innate response could be related, at least to some extent, to a differences in host density among different fruit species in nature [64,68,69]. Field and laboratory studies showed that the most frequent host fruit for *Bactrocera* species are mango (*Mangifera indica* L.), papaya (*Carica papaya* L.), guava (*Psidium guajava* L.), several species of *Cucurbita*, as well as native species of varying taxonomic families (e.g. *Psidium* sp., *Ziziphus* sp., *Syzypium* sp., etc.). Infestation of *Citrus* spp. by *Bactrocera* is rare [51,70,71]. Moreover, a survey performed of fruit flies (Diptera: Tephritidae) from wild and cultivated host plants species in Thailand and Malaysia found that *D*. *longicaudata* was frequently recovered from several species of Moraceae family (the same family of fig) [51]. According to White and Elson-Harris [52], apple is not an important host for *Bactrocera spp*. and only 10% of the species of this genus attack this fruit species. Although it is not possible to directly link these finding to the innate preference of *D*. *longicaudata* found in our experiments, a differential distribution of the native hosts among fruit species could account for the preference for fig over apple and orange.

The innate preference of *D*. *longicaudata* could also be related to differences among fruit species in the available enemy-free space [72]. Parasitism rates on fig were higher than those found on peach and orange. This might be related to the smaller size of figs which would disable larvae to escape from parasitism [73,74]. These differences could therefore explain the preference of *D*. *longicaudata* for fig within the olfactometer, but on the other hand no preference should be expected between peach and orange (on which the parasitism rate was not different), and olfactometer results showed a clear preference for peach. (Comparisons of parasitism rates between apple and the rest of the fruit species are limited because of its lower number of larvae per cm^2^). The association between preference and realized parasitism rate has been studied in *D*. *longicaudata* by Leyva et al. [53] and Ovruski et al. [57]. Altogether, results so far seem to indicate no clear association between the preferred host fruit and the parasitism rate, at least not an association that completely explains the preference for host habitats.

Several studies have focused on the PPH at the bitrophic scale, but the connection between preference and performance considering the first and third trophic level has seldom been studied [45]. In the present study, we did not detect a clear association between the preferences of female parasitoids for certain host habitats and their reward in terms of fitness. In fact fecundity and sex ratio showed that performance was higher for parasitoids associated to oranges, while survival was not affected by fruit species. This is in agreement with results obtained by Eben et al. [48] (who compared oranges and mangoes), which together with our results suggest that orange would be a fruit that produces high quality hosts even when it is not the preferred host habitat. This lack of a perfect association between preference and performance when the first and third trophic levels are considered seems to be quite frequent [46,48,53,75] with cases in which PPH is supported being rare [45]. It could be argued that the correspondence between preference and performance fades away as the distance between trophic levels increases, and other factors, such as realized parasitism rate and the oviposition preference of the adult flies, might contribute to a larger extent to the parasitoid preference. This idea needs further research.

The size of the herbivore is a good estimator of its quality as host for koinobiont parasitoids that attack late instar larvae, like *D*. *longicaudata* [76–80]. We found an effect of the host fruit on the weight of *C*. *capitata* pupae, with apple showing the lowest weight. Similar results were obtained for adult size, which is considered an important component of parasitoid fitness [18,19, 81]. This could explain the fact that apple was the least chosen fruit within the olfactometer. Nonetheless, these parameters cannot entirely explain the pattern of preference for different fruits, because pupae that developed in peach and orange showed similar weights and adult size, with fig taking intermediate values. Again, preference for host habitats and performance associated to each one of them are not clearly correlated.

Despite an innate preference for host habitats, we found that *D*. *longicaudata* preference is modulated by the level of infestation (patch richness) and the experience acquired during foraging. *Diachasmimorpha longicaudata* is able to discriminate between infested and non-infested fruit based on infochemicals [48,82–84]. Our results showed that females are also capable of discerning between different levels of infestations, and orientate themselves to the more profitable patches. Moreover, chemical information about host abundance overrides the preference for a given fruit. This suggests that when females have a reliable cue of the host presence, orientation behaviour will no longer respond to innate preferences, something that has been documented in other host-parasitoid systems [13,85–87]. By doing so, parasitoids can adjust their host searching strategy and seek those habitats in which the probability of host encounter is higher.

Previous experience also affected the host habitat preference of *D*. *longicaudata*. This shows again that *D*. *longicaudata* can assimilate information about the distribution of its hosts and modify accordingly their foraging behaviour, in this case probably mediated by associative learning (because in the olfactometer trials fruit was not infested). Learning of chemical cues by insect parasitoids has been reported in several species and behavioural contexts, such as food [88–90] or host finding [14,91,92]. The effect of learning is so drastic for this species that it generates a preference for colours when there is no innate preference for such cue [60] and, as shown here, blurs the innate pattern of preference for fruit species based on chemical cues.

Our results provide information about the host finding behaviour and, consequently, the effectiveness of *D*. *longicaudata* as a biological control agent of Tephritidae fruit fly pests. We demonstrated that female parasitoids can use chemical information both from the host and the host habitat during the host searching process. Females will orientate preferentially to specific host habitats in the absence of direct cues from their hosts, which would allow to reduce the searchable area and increases the probability of host encounter. However, if direct cues are perceived, females will orientate to the infested fruits [83] apparently in a dose-response manner, as they are able to detect different infestation levels. After finding suitable hosts, females are able to associate conspicuous chemical and visual cues from these host habitats that can be used in successive foraging bouts. The plasticity in the use of chemical and visual cues makes this species a good candidate to control hosts that can be encountered in fruits of different species, which are ephemeral habitats that change markedly in their chemical and physical properties along the season.

## Supporting Information

S1 Table Latency times (mean ± S.E.) recorded in the Y-tube olfactometer in experiment 1. (DOCX)

S2 Table Latency times (mean ± S.E.) recorded in the Y-tube olfactometer in experiment 2. (DOCX)

S3 Table Latency times (mean ± S.E.) for the selection in the Y-tube olfactometer in experiment 3. (DOCX)

## References

[1] Turlings TC, Loughrin JH, Mccall PJ, Röse US, Lewis W J, Tumlinson J H. How caterpillar-damaged plants protect themselves by attracting parasitic wasps. Proc Natl Acad Sci. 1995; 92: 4169–4174. 7753779 10.1073/pnas.92.10.4169 PMC41905

[2] de Moraes CM, Lewis WJ, Pare PW, Alborn HT, Tumlinson JH. Herbivore-infested plants selectively attract parasitoids. Nature. 1998; 393: 570–573.

[3] Dicke M, Vet LEM. Plant-carnivore interactions: evolutionary and ecological consequences for plant, herbivore and carnivore In: Olff H, Brown VK, Drent RH editors. Herbivores: between plants and predators. Oxford, UK: Blackwell Science; 1999 pp. 483–520.

[4] Turlings TCJ, Tumlinson JH, Lewis WJ. Exploitation of herbivore-induced plant odors by host seeking parasitic wasps. Science. 1990; 250: 1251–1253. 17829213 10.1126/science.250.4985.1251

[5] Cortesero AM, De Moraes CM, Stapel JO, Tumlinson JH, Lewis WJ. Comparisons and contrasts in host-foraging strategies of two larval parasitoids with different degrees of host specificity. J Chem Ecol. 1997; 23: 1589–1606.

[6] Ngumbi E, Chen L, Fadamiro HY. Comparative GC-EAD responses of a specialist (*Microplitis croceipes*) and a generalist (*Cotesia marginiventris*) parasitoid to cotton volatiles induced by two caterpillar species. J Chem Ecol. 2009; 35: 1009–1020. 10.1007/s10886-009-9700-y 19802643

[7] Turlings TC, Wäckers F. Recruitment of predators and parasitoids by herbivore-injured plants. Adv Insect Chem Ecol. 2004; 2: 21–75.

[8] Dicke M. Behavioural and community ecology of plants that cry for help. Plant Cell Environ. 2009; 32: 654–665. 10.1111/j.1365-3040.2008.01913.x 19021885

[9] Dicke M, Baldwin IT. The evolutionary context for herbivore-induced plant volatiles: beyond the ‘cry for help’. Trends Plant Sci. 2010; 15: 167–175. 10.1016/j.tplants.2009.12.002 20047849

[10] Hoffmeister TS, Gienapp P. Exploitation of the host’s chemical communication in a parasitoid searching for concealing host larvae. Ethology. 1999; 105:223–232.

[11] Vet LEM, Dicke M. Ecology of infochemical use by natural enemies in a tritrophic context. Annu Rev Entomol. 1992; 37: 141–172.

[12] Turlings TCJ, Wäckers FL, Vet LEM, Lewis WJ, Tumlinson JH. Learning of host-finding cues by hymenopterous parasitoids In: Papaj DR, Lewis AC, editors. Insect Learning. New York: Chapman y Hall; 1993 pp. 51–78.

[13] Geervliet JBF, Vreugdenhil AI, Dicke M, Vet EM. Learning to discriminate between infochemicals from different plant-host complexes by the parasitoids *Cotesia glomerata* and *C*. *rubecula*. Entomol Exp Appl. 1998; 86: 241–252.

[14] Stireman JO III. Learning in the generalist tachinid parasitoid *Exorista Mella* Walker (Diptera: Tachinidae). J Insect Behav. 2002; 15: 689–706.

[15] Takemoto H, Powell W, Pickett J, Kainoh Y, Takabayashi J. Two-step learning involved in acquiring olfactory preferences for plant volatiles by parasitic wasps. Anim Behav. 2012; 83: 1491–1496.

[16] Steidle JL, Van Loon JJ. Dietary specialization and infochemical use in carnivorous arthropods: testing a concept. Entomol Exp Appl. 2003; 108: 133–148.

[17] Godfray HCJ. Parasitoids: behavioral and evolutionary ecology Princeton, USA Princenton University Press; 1994.

[18] Harvey JA. Factors affecting the evolution of development strategies in parasitoid wasps: the importance of functional constraints and incorporating complexity. Ent Exp Appl. 2005; 117: 1–13.

[19] Bukovinszky T, Gols R, Smid HM, Bukovinszkiné Kiss G, Dicke M, Harvey JA. Consequences of constitutive and induced variation in the host’s food plant quality for parasitoid larval development. J Insect Physiol. 2012; 58: 367–375. 10.1016/j.jinsphys.2011.12.017 22233934

[20] Stephens DW, Krebs JR. Foraging theory Princeton: University Press; 1986.

[21] Charnov EL, Stephens DW. On the evolution of host selection in solitary parasitoids. Am Nat. 1988; 132: 707–722.

[22] Harvey JA, Gols R, Snaas H, Malcicka M, Visser B. Host preference and offspring performance are linked in three congeneric hyperparasitoid species. Ecol Entomol. 2015; 40: 114–122.

[23] Levins R, MacArthur RH. An hypothesis to explain the incidence of monophagy. Ecology. 1969; 50: 9l0–911.

[24] Jaenike J. On optimal oviposition behaviour in phytophagous insects, Theor. Popul. Biol. 1978; 14:350–356. 751265 10.1016/0040-5809(78)90012-6

[25] Thompson JN. Coevolution and alternative hypotheses on insect/plant interactions. Ecology. 1988; 69: 893–895.

[26] Mayhew PJ. Adaptive patterns of host-plant selection by phytophagous insects. Oikos. 1997; 79: 417–428.

[27] Mayhew PJ. The evolution of gregariousness in parasitoid wasps. Proc R Soc Lond B. 1998; 265: 383–389

[28] Gripenberg S, Mayhew PJ, Parnell M, Roslin T. A meta‐analysis of preference–performance relationships in phytophagous insects. Ecol Lett. 2010; 13: 383–393. 10.1111/j.1461-0248.2009.01433.x 20100245

[29] van Alphen JJM, Vet LEM. An evolutionary approach to host finding and selection In: Waage JK, Greathead D, editors. Insect Parasitoids London: Academic Press; 1986 pp. 23–61.

[30] Rivero A. The relationship between host selection behaviour and offspring fitness in a koinobiont parasitoid. Ecol Entomol. 2000; 25: 467–472.

[31] Luhring KA, Millar JG, Paine TD, Reed D, Hanks LM, Christiansen H. Oviposition preferences and progeny development of the egg parasitoid *Avetianella longoi*: factors mediating replacement of one species by a congener in a shared habitat. Biol Control. 2004; 30: 382–391.

[32] Ode PJ, Berenbaum MR, Zangerl AR, Hardy ICW. Host plant, host plant chemistry and the polyembryonic parasitoid *Copidosoma sosares*: indirect effects in a tritrophic interaction. Oikos. 2004; 104: 388–400.

[33] Cicero L, Sivinski J, Aluja M. Effect of host diet and adult parasitoid diet on egg load dynamics and egg size of braconid parasitoids attacking *Anastrepha ludens*. Physiol Entomol. 2012; 37: 177–184.

[34] Jandricic SE, Dale AG, Bader A, Frank SD. The effect of banker plant species on the fitness of *Aphidius colemani* Viereck and its aphid host (*Rhopalosiphum padi* L.). Biol Control. 2014; 76: 28–35.

[35] Li X, Niu Y, Liu T. The performance of *Oomyzus sokolowskii* (Hymenoptera: Eulophidae) parasitizing *Plutella xylostella* (Lepidoptera: Plutellidae) on different host plants. Appl Entomol Zool. 2014; 49: 67–75.

[36] Benrey B, Denno RF. The slow-growth—high-mortality hypothesis: A test using the cabbage butterfly. Ecology. 1997; 78: 987–999.

[37] Ode PJ. Plant chemistry and natural enemy fitness: Effects on herbivore and natural enemy interactions. Annu Rev Entomol. 2006; 51:163–85. 16332208 10.1146/annurev.ento.51.110104.151110

[38] Gols R, Bukovinszky T, van Dam NM, Dicke M, Bullock JM, Harvey JA. Performance of generalist and specialist herbivores and their endoparasitoids differs on cultivated and wild *Brassica* populations. J Chem Ecol. 2008; 34: 132–143. 10.1007/s10886-008-9429-z 18231835 PMC2239250

[39] Blaul B, Ruther J. How parasitoid females produce sexy sons: a causal link between oviposition preference, dietary lipids and mate choice in Nasonia. Proc. R. Soc. B. 2011; 278: 3286–3293. 10.1098/rspb.2011.0001 21429922 PMC3169019

[40] Harvey JA, Gols R. Population-related variation in plant defense more strongly affects survival of an herbivore than its solitary parasitoid wasp. J Chem Ecol. 2011; 37: 1081–1090. 10.1007/s10886-011-0024-3 21987026 PMC3197929

[41] Stoepler TM, Lill JT, Murphy SM. Cascading effects of host size and host plant species on parasitoid resource allocation. Ecol Entomol. 2011; 36: 724–735.

[42] Rutledge CE, Wiedenmann RN. Habitat preferences of three congeneric braconid parasitoids: implications for host-range testing in biological control. Biol Control. 1999; 16: 144–154.

[43] Wyckhuys KAG, Heimpel GE. Response of the soybean aphid parasitoid *Binodoxys communis* to olfactory cues from target and non-target host-plant complexes. Entomol Exp Appl. 2007; 132: 149–158.

[44] Belda C, Riudavets J. Attraction of the parasitoid *Anisopteromalus calandrae* (Howard) (Hymenoptera: Pteromalidae) to odors from grain and stored product pests in a Y-tube olfactometer. Biol Control. 2010; 54: 29–34.

[45] Gols R, Harvey JA. Plant-mediated effects in the Brassicaceae on the performance and behaviour of parasitoids. Phytochem Rev. 2009; 8: 187–206.

[46] Fathi SAA, Bozorg-Amirkalaee M, Sarfraz RM, Rafiee-Dastjerdi H. Parasitism and developmental parameters of the parasitoid *Diadegma majale* (Gravenhorst) in control of *Plutella xylostella* (L.) on selected cultivars of canola. BioControl. 2012; 57: 49–59.

[47] Sarfraz M, Dosdall LM, Keddie BA. Influence of the herbivore host’s wild food plants on parasitism, survival and development of the parasitoid *Diadegma insulare*. Biol Control. 2012; 62: 38–44.

[48] Eben A, Benrey B, Sivinski J, Aluja M. Host species and host plant effects on preference and performance of *Diachasmimorpha longicaudata* (Hymenoptera: Braconidae). Environ Entomol. 2000; 29: 87–94.

[49] Ero MM, Clarke AR. Host location by the fruit fly parasitoid *Diachasmimorpha krausii*: role of fruit fly species, life stage and host plant. Agric For Entomol. 2012; 14: 101–110.

[50] Wharton RA, Gilstrap FE. Key to and status of Opiinae Braconid (Hymenoptera) parasitoids used in biological control of *Ceratitis* and *Dacus* s.l. (Diptera: Tephritidae). Ann Entomol Soc Am. 1983; 76: 721–742.

[51] Chinajariyawong A, Clarke AR, Jirasurat M, Kritsaneepiboon S, Lahey HA, Vijaysegaran S, et al Survey of opiineparasitoids of fruit flies (Diptera: Tephritidae) in Thailand and Malaysia. The Raffles Bulletin of Zoology. 2000; 48: 71–101.

[52] White IM, Elson-Harris MM. Fruit flies of economic significance; their identification and bionomics Wallingford: CAB International; 1992.

[53] Leyva JL, Browning HW, Gilstrap FE. Effect of host fruit species, size, and colour on parasitization of *Anastrepha ludens* (Diptera: Tephritidae) by *Diachasmimorpha longicaudata* (Hymenoptera: Braconidae). Environ Entomol. 1991; 20: 1469–1474.

[54] Messing RH, Jang EB. Response of the fruit fly parasitoid *Diachasmimorpha longicaudata* (Hymenoptera: Braconidae) to host-fruit stimuli. Environ Entomol. 1992; 21: 1189–1195.

[55] Ovruski SM, Oroño LE, Schliserman P, Nuñez-Campero S. The effect of four fruit species on the parasitization rate of *Anastrepha fraterculus* (Diptera: Tephritidae, Trypetinae) by *Diachamimorpha longicaudata* (Hymenoptera: Braconidae, Opiinae) under laboratory conditions. Biocontrol Sci Techn. 2007; 17: 1079–1085.

[56] Cicero L, Sivinski J, Rull J, Aluja M. Effect of larval host food substrate on egg load dynamics, egg size and adult female size in four species of braconid fruit fly (Diptera: Tephritidae) parasitoids. J Insect Physiol. 2011; 57: 1471–1479. 10.1016/j.jinsphys.2011.07.014 21819991

[57] Ovruski SM, van Nieuwenhove G, Bezdjian L, Albornoz-Medina P, Schliserman P. Evaluation of *Diachasmimorpha longicaudata* (Hymenoptera: Braconidae) as a mortality factor of *Ceratitis capitata* (Diptera: Tephritidae) infesting *Citrus* species under laboratory and field-cage conditions. Biocontrol Sci Techn. 2012; 22: 187–202.

[58] Dukas R, Duan JJ. Potential fitness consequences of associative learning in a parasitoid wasp. Behav Ecol. 2000; 11: 536–543.

[59] Benelli G, Canale A. Learning of visual cues in the fruit fly parasitoid *Psyttalia concolor* (Szépligeti) (Hymenoptera: Braconidae). BioControl. 2012; 57: 767–777. 10.1017/S0007485312000715 23302745

[60] Segura DF, Viscarret MM, Carabajal Paladino LZ, Ovruski SM, Cladera JL. Role of visual information and learning in habitat selection by a generalist parasitoid foraging for concealed hosts. Anim Behav. 2007; 74: 131–142.

[61] Birke A, Aluja M. *Anastrepha ludens* and *Anastrepha serpentina* (Diptera: Tephritidae) do not infest *Psidium guajava*, but *Anastrepha obliqua* occasionally shares this resource with *Anastrepha striata* in nature. J Econ Entomol. 2011; 104: 1204–1211. 21882684 10.1603/ec11042

[62] Devescovi F, Liendo MC, Bachmann GE, Bouvet JP, Milla FH, Vera MT, et al Fruit infestation patterns by *Anastrepha fraterculus* and *Ceratitis capitata* reveal that cross-recognition does not lead to complete avoidance of interspecific competition in nature. Agric For Entomol. 2015; 17: 325–335.

[63] Viscarret MM, La Rossa R, Segura DF, Ovruski SM, Cladera JL. Evaluation of the parasitoid *Diachasmimorpha longicaudata* (Ashmead) (Hymenoptera: Braconidae) reared on a genetic sexing strain of *Ceratitis capitata* (Wied.) (Diptera: Tephritidae). Biol Control. 2006; 36: 147–153.

[64] Ovruski SM, Colin C, Soria A, Oroño L, Schliserman P. Introducción y establecimiento en laboratorio de *Diachasmimorpha tryoni* y *Diachasmimorpha longicaudata* (Hymenoptera: Braconidae, Opiinae) para el control biológico de *Ceratitis capitata* (Diptera: Tephritidae, Dacinae) en la Argentina. Rev Soc Entomol Argent. 2003; 62:49–59.

[65] Terán H. Comportamiento alimentario y su correlación a la reproducción en hembras de *Ceratitis capitata* Wied. Rev Agron del NOA. 1977; 14: 17–34.

[66] Segura DF, Vera MT, Cladera JL. Dinámica de la infestación por estadios inmaduros de la mosca del Mediterráneo, *Ceratitis capitata* (Diptera: Tephritidae), en San Pedro, pcia. de Buenos Aires. Ecología Austral. 2004; 14: 3–17.

[67] StatSoft, Inc. STATISTICA for Windows (program manual). Tulsa OK, USA 2000.

[68] Mavrikakis PG, Economopoulos AP, Carey JR. Continuous winter reproduction and growth of the Mediterranean fruit fly (Diptera: Tephritidae) in Heraklion, Crete, Southern Greece. Environ Entomol. 2000; 29: 1180–1187.

[69] Segura DF, Vera MT, Cagnotti C, Vaccaro N, de Coll O, Ovruski SM, et al Relative abundance of *Ceratitis capitata* and *Anastrepha fraterculus* (Diptera: Tephritidae) in diverse host species and localities of Argentina. Ann Entomol Soc Am. 2006; 99: 70–83.

[70] Clarke AR, Armstrong KF, Carmichael AE, Milne JR, Raghu S, Roderick GK, et al Invasive phytophagous pests arising through a recent tropical evolutionary radiation: The *Bactrocera dorsalis* complex of fruit flies. Annu Rev Entomol. 2005; 50: 293–319. 15355242 10.1146/annurev.ento.50.071803.130428

[71] Rwomushana I, Ekesi S, Gordon I, Ogol CKPO. Host plants and host plant preference studies for *Bactrocera invadens* (Diptera: Tephritidae) in Kenya, a new invasive fruit fly species in Africa. Ann Entomol Soc Am. 2008; 101: 331–340.

[72] Denno RF, Larsson S, Olmstead KL. Host plant selection in willow-feeding leaf beetles (Coleoptera: Chrysomelidae): role of enemy-free space and plant quality. Ecology. 1990; 71: 124–137.

[73] Sivinski J. The influence of host fruit morphology on parasitization rates in the Caribbean fruit fly, *Anastrepha suspensa*. Entomophaga. 1991; 36: 447–454.

[74] Suárez L, Murúa F, Lara N, Escobar J, Taret G, Rubio J, et al Biological control of *Ceratitis capitata* (Diptera: Tephritidae) in Argentina: releases of *Diachasmimorpha longicaudata* (Hymenoptera: Braconidae) in fruit-producing semi-arid areas of San Juan. Nat Sci. 2014; 6: 664–675.

[75] Ero MM, Hamacek E, Clarke A. Foraging behaviours of *Diachasmimorpha kraussii* (Fullaway) (Hymenoptera: Braconidae) and its host *Bactrocera tryoni* (Froggatt) (Diptera: Tephritidae) in a nectarine (*Prunus persica* (L.) Batsch var. nectarina (Aiton) Maxim) orchard. Aust J Entomol. 2011; 50: 234–240.

[76] Harvey JA, Harvey IF, Thompson DJ. Flexible larval growth allows use of a range of host sizes by a parasitoid wasp. Ecology. 1994; 75: 1420–1428.

[77] Croft P, Copland MJ. The effect of host instar on the size and sex ratio of the endoparasitoid *Dacnusa sibirica*. Entomol Exp Appl. 1995; 74: 121–124.

[78] Harvey JA, Bezemer TM, Elzinga JA, Strand MR. Development of the solitary endoparasitoid *Microplitis demolitor*: host quality does not increase with host age and size. Ecol Entomol. 2004; 29: 35–43.

[79] Haeckermann J, Rott AS, Dorn S. How two different host species influence the performance of a gregarious parasitoid: host size is not equal to host quality. J Anim Ecol. 2007; 76: 376–383. 17302845 10.1111/j.1365-2656.2006.01206.x

[80] Sequeira R, Mackauer M. Nutritional ecology of an insect host-parasitoid association: the pea-aphid *Aphidus ervi* system. Ecology. 1992; 73: 183–189.

[81] Visser ME. The importance of being large: the relationship between size and fitness in females of the parasitoid *Aphaereta minuta* (Hymenoptera: Braconidae). J Anim Ecol. 1994; 63: 963–978.

[82] Silva JWP, Bento JMS, Zucchi RA. Olfactory response of three parasitoid species (Hymenoptera: Braconidae) to volatiles of guavas infested or not with fruit fly larvae (Diptera: Tephritidae). Biol Control. 2007; 41: 304–311.

[83] Segura DF, Viscarret MM, Ovruski SM, Cladera JL. Response of the fruit fly parasitoid *Diachasmimorpha longicaudata* to host and host-habitat volatile cues. Entomol Exp Appl. 2012; 143: 164–176.

[84] Carrasco M, Montoya P, Cruz-López L, Rojas JC. Responses of the fruit fly parasitoid *Diachasmimorpha longicaudata* (Hymenoptera: Braconidae) to mango fruit volatiles. Environ Entomol. 2005; 34: 576–583.

[85] Tentelier C, Wajnberg E, Fauvergue X. Parasitoids use herbivore-induced information to adapt patch exploitation behaviour. Ecol Entomol. 2005; 30: 739–744.

[86] Tentelier C, Fauvergue X. Herbivore-induced plant volatiles as cues for habitat assessment by a foraging parasitoid. J Anim Ecol. 2007; 76: 1–8. 17184347 10.1111/j.1365-2656.2006.01171.x

[87] Uefune M, Nakashima Y, Tagashira E, Takabayashi J, Takagi M. Response of *Wollastoniella rotunda* (Hemiptera: Anthocoridae) to volatiles from eggplants infested with its prey *Thrips palmi* and *Tetranychus kanzawai*: prey species and density effects. Biol Control. 2010; 54: 19–22.

[88] Lewis WJ, Takasu K. Use of learned odours by a parasitic wasp in accordance with host and food needs. Nature. 1990; 348: 635–636.

[89] Patt JM, Hamilton GC, Lashomb JH. Response of two parasitoid wasps to nectar odors as a function of experience. Entomol Exp Appl. 1999; 90: 1–8.

[90] Sato M, Takasu K. Food odor learning by both sexes of the pupal parasitoid *Pimpla alboannulatus* Uchida (Hymenoptera: Ichneumonidae). J Insect Behav. 2000; 13: 263–272.

[91] Wäckers FL, Lewis WJ. Olfactory and visual learning and their combined influence on host site location by the parasitoid *Microplitis croceipes* (Cresson). Biol Control. 1994; 4: 105–112.

[92] Kerguelen V, Cardé RT. Flight toward a learned odor and factors inducing landing of female *Brachymeria intermedia* (Hymenoptera: Chalcididae), a parasitoid of the gypsy moth, *Lymantria dispar* (Lepidoptera: Lymantriidae). J Insect Behav. 1998; 11: 221–234.

