# Supplementary material for: Innate Host Habitat Preference in the Parasitoid Diachasmimorpha longicaudata: Functional Significance and Modifications through Learning
Source: PLoS One. 2016 Mar 23;11(3):e0152222. doi: 10.1371/journal.pone.0152222 (PMC4805301; doi:10.1371/journal.pone.0152222)
Supplement: S1 Table — (DOCX) [file pone.0152222.s001.docx]

**S1 Table. Latency times (mean ± S.E.) recorded in the Y-tube olfactometer in experiment 1.**

| Pair-wise fruit combination | | Latency (in s) | | t _1,38_ | | P-value |  |  |
| --- | --- | --- | --- | --- | --- | --- | --- | --- |
| Peach vs. fig | P: 369.975 ± 20.669  F: 355.543 ± 38.621 | | 0.357 | | 0.722 | | |  |
| Peach vs. apple | P: 402.536 ± 18.340  A: 421.355 ± 32.467 | | 0.468 | | 0.642 | | |  |
| Peach vs. orange | P: 364.979 ± 26.618  O: 385.581 ± 36.143 | | 0.437 | | 0.664 | | |  |
| Fig vs. apple | F: 324.897 ± 14.784  A: 340.630 ± 53.386 | | 0.361 | | 0.720 | | | |
| Fig vs. orange | F: 333.325 ± 16.597  O: 419.408 ± 33.208 | | 2.584 | | 0.014 | | | |
| Apple vs. orange | A: 375.737 ± 27.164  O: 388.243 ± 15.585 | | 0.413 | | 0.682 | | | |

Parameters from the student *t*-test (t, p-value) are also presented.

A: apple, F: fig, O: orange, P: peach.
