# Supplementary material for: Innate Host Habitat Preference in the Parasitoid Diachasmimorpha longicaudata: Functional Significance and Modifications through Learning
Source: PLoS One. 2016 Mar 23;11(3):e0152222. doi: 10.1371/journal.pone.0152222 (PMC4805301; doi:10.1371/journal.pone.0152222)
Supplement: S2 Table — (DOCX) [file pone.0152222.s002.docx]

**S2 Table. Latency times (mean ± S.E.) recorded in the Y-tube olfactometer in experiment 2.**

| Pair-wise fruit combination | Latency (in s) | t _1,38_ | P-value |
| --- | --- | --- | --- |
| Apple H vs. Apple -L | A-H: 374.853 ± 18.590  A-L: 431.555 ± 36.738 | 1.367 | 0.180 |
| Orange H vs. Orange L | O-H: 413.805 ± 18.583  O-L: 394.368 ± 36.975 | 0.522 | 0.604 |
| Orange H vs. Apple L | O-H: 307.144 ± 23.531  A-L: 366.524 ± 44.352 | 1.031 | 0.310 |
| Apple H vs. Orange L | A-H: 375.764 ± 25.562  O-L: 339.627 ± 23.697 | 1.025 | 0.312 |

Parameters from the student *t*-test (t, p-value) are also presented.

A: apple, O: orange, H: high infestation level, L: low infestation level.
