# Supplementary material for: Innate Host Habitat Preference in the Parasitoid Diachasmimorpha longicaudata: Functional Significance and Modifications through Learning
Source: PLoS One. 2016 Mar 23;11(3):e0152222. doi: 10.1371/journal.pone.0152222 (PMC4805301; doi:10.1371/journal.pone.0152222)
Supplement: S3 Table — (DOCX) [file pone.0152222.s003.docx]

**S3 Table. Latency times (mean ± S.E.) for the selection in the Y-tube olfactometer in experiment 3.**

| Previous experience | Latency (in s) | t _1,38_ | P |
| --- | --- | --- | --- |
| Conditioned in apple | A: 383.974 ± 31.127  O: 429.918 ± 29.886 | 0.890 | 0.379 |
| Conditioned in orange | A: 369.054 ± 67.182  O: 415.13 ± 11.8402 | 0.923 | 0.362 |

Parameters from the student *t*-test (t, p-value) are also presented. A: Apple, O: Orange.
